# Predicting BRAFV600E mutations in papillary thyroid carcinoma using six machine learning algorithms based on ultrasound elastography

**DOI:** 10.1038/s41598-023-39747-6

**Published:** 2023-08-03

**Authors:** Enock Adjei Agyekum, Yu-guo Wang, Fei-Ju Xu, Debora Akortia, Yong-zhen Ren, Kevoyne Hakeem Chambers, Xian Wang, Jenny Olalia Taupa, Xiao-qin Qian

**Affiliations:** 1 Ultrasound Medical Laboratory, Department of Ultrasound, Affiliated People’s Hospital of Jiangsu University, Zhenjiang, 212002 China; 2 School of Medicine, Jiangsu University, Zhenjiang, 212013 China; 3 Department of Ultrasound, Traditional Chinese Medicine Hospital of Nanjing Lishui District, Nanjing, China; 4 School of Public Health, Kwame Nkrumah University of Science and Technology, Kumasi, Ghana

**Keywords:** Medical research, Oncology

## Abstract

The most common BRAF mutation is thymine (T) to adenine (A) missense mutation in nucleotide 1796 (T1796A, V600E). The BRAF^V600E^ gene encodes a protein-dependent kinase (PDK), which is a key component of the mitogen-activated protein kinase pathway and essential for controlling cell proliferation, differentiation, and death. The BRAF^V600E^ mutation causes PDK to be activated improperly and continuously, resulting in abnormal proliferation and differentiation in PTC. Based on elastography ultrasound (US) radiomic features, this study seeks to create and validate six distinct machine learning algorithms to predict BRAF^V6OOE^ mutation in PTC patients prior to surgery. This study employed routine US strain elastography image data from 138 PTC patients. The patients were separated into two groups: those who did not have the BRAF^V600E^ mutation (n = 75) and those who did have the mutation (n = 63). The patients were randomly assigned to one of two data sets: training (70%), or validation (30%). From strain elastography US images, a total of 479 radiomic features were retrieved. Pearson's Correlation Coefficient (PCC) and Recursive Feature Elimination (RFE) with stratified tenfold cross-validation were used to decrease the features. Based on selected radiomic features, six machine learning algorithms including support vector machine with the linear kernel (SVM_L), support vector machine with radial basis function kernel (SVM_RBF), logistic regression (LR), Naïve Bayes (NB), K-nearest neighbors (KNN), and linear discriminant analysis (LDA) were compared to predict the possibility of BRAF^V600E^. The accuracy (ACC), the area under the curve (AUC), sensitivity (SEN), specificity (SPEC), positive predictive value (PPV), negative predictive value (NPV), decision curve analysis (DCA), and calibration curves of the machine learning algorithms were used to evaluate their performance. ① The machine learning algorithms' diagnostic performance depended on 27 radiomic features. ② AUCs for NB, KNN, LDA, LR, SVM_L, and SVM_RBF were 0.80 (95% confidence interval [CI]: 0.65–0.91), 0.87 (95% CI 0.73–0.95), 0.91(95% CI 0.79–0.98), 0.92 (95% CI 0.80–0.98), 0.93 (95% CI 0.80–0.98), and 0.98 (95% CI 0.88–1.00), respectively. ③ There was a significant difference in echogenicity,vertical and horizontal diameter ratios, and elasticity between PTC patients with BRAF^V600E^ and PTC patients without BRAF^V600E^. Machine learning algorithms based on US elastography radiomic features are capable of predicting the likelihood of BRAF^V600E^ in PTC patients, which can assist physicians in identifying the risk of BRAF^V600E^ in PTC patients. Among the six machine learning algorithms, the support vector machine with radial basis function (SVM_RBF) achieved the best ACC (0.93), AUC (0.98), SEN (0.95), SPEC (0.90), PPV (0.91), and NPV (0.95).

## Introduction

The BRAF^V600E^ mutation is a significant contributor to the papillary thyroid carcinoma (PTC) phenotype, which aids in the diagnosis and differential diagnoses of PTC before surgery^1,2^. BRAF^V600E^ diagnosis requires genetic testing of cell eluate by ultrasound-guided fine-needle aspiration (FNA), which is invasive. Ultrasound-guided FNA cytological examination of thyroid nodules can diagnose PTC before surgery, but 15% to 30% of the cytological results belong to the Bethesda system definition with uncertain detection results (including Bethesda Type III: atypical lesions or follicular lesions of unknown significance (AUS/FLUS), Type IV: follicular tumors/suspected follicular tumors, and Type V: suspected malignant tumors (SUSP)). Therefore, "TBSRTC Classification Malignant Risk and Management Recommendation" recommends FNA cytology combined with BRAF^V600E^ mutation detection, but are all invasive. As a result, it is critical in clinical practice to adopt non-invasive approaches to forecast the status of BRAF^V600E^ mutations, so as to reduce the FNA and molecular detection rate.

More importantly, According to earlier research, BRAF^V600E^ mutation in thyroid tumors is thought to be a sign of severe illness and PTC-related mortality^3^. It is interesting to note that the presence of the BRAF^V600E^ mutation has become a more reliable molecular marker for PTC recurrence^3^. Therefore, finding BRAF^V600E^ mutations in thyroid tumors has implications for prognosis and serves as a marker for tumor recurrence. The BRAF^V600E^ mutation is also strongly linked to the existence of extrathyroidal extension (ETE) and cervical lymph node metastasis (CLNM) in PTC patients, suggesting an invasion^4,5^. Jin et al.^6^ observed a substantial relationship between BRAF^V600E^ mutation with CLNM and ETE in a Mayo Clinic research. Xing et al.^7^ revealed a close link between BRAF^V600E^ mutation and ETE, CLNM, and advanced illness stages in a large comprehensive international multicenter investigation. Even if either gene mutation is capable of identifying aggressive cancer types, the genetic analysis still requires specimen tissue for examination, which is often collected by invasive surgical procedures. Furthermore, identifying tumors using FNA biopsy samples from type 4a nodules is difficult because tumor cells vary in quantity, quality, and purity^8^. A sensitive and precise BRAF^V600E^ mutation detection method will thus aid in the early detection of PTC^9^. Because BRAF^V600E^ gene mutations produce 99.8% of malignant nodules, it is a significant tumor marker for PTC.

With the increasing popularity of tumor thermal ablation technology (microwave, radio frequency, and laser) in China, an increasing number of patients with PTC, particularly those with minimal papillary thyroid cancer (MPTC), are willing to accept thermal ablation as a minimally invasive procedure to maximize the preservation of thyroid function. As a result, proper preoperative detection of BRAF^V600E^ status in PTC patients is critical for patients to choose therapy methods. Surgery is currently chosen over ablation for highly invasive PTC in China.

The main imaging method for evaluating thyroid nodules is ultrasound (US)^10^. Grayscale US has recently been found to be able to predict the mutation of BRAF^V600E^ in PTC. However, the findings are still up for debate^11,12^, which may be related to the drawbacks of the conventional US image, such as its dependence on the radiologist's experience and interobserver variation^13^. The ability of a tissue to resist deformation whenever a force is exerted on it or to restore its initial form when that force is withdrawn is called elasticity, which is what elastography often examines in tissues. Depending on the Doppler US technology, strain images can be presented in grayscale or in colors that reflect the stiffness and elasticity of the tissues^14–17^. Elastography is also based on the grayscale US which is also subjective and operator dependent. There have been several significant developments to increase the performance of US elastography such as elastography estimation from Doppler imaging using central difference and least-squares algorithms^17^.

Radiomics analysis using US images has been used to forecast the molecular characteristics of several malignancies, which include PTC^18,19^. Artificial intelligence (AI) has considerably expanded in recent years as a cutting-edge data analysis tool in the medical field^20^. As a result of its extensive digital data sets, radiology in particular is well suited for AI^21^. Recently, there has been a lot of interest in the medical area in the use of radiomics in conjunction with machine learning, which is an important subset of AI and plays a tremendous supporting role in increasing diagnostic and prognostic accuracy^22,23^. However, there are currently few studies that used machine learning models primarily on elastography US radiomics data to detect the existence of BRAF^V6OOE^ mutation in PTC. There has also been no research that employed different machine learning classifiers in assessing BRAF^V6OOE^ in PTC patients using elastography US radiomic features. Based on elastography US radiomic features, this study seeks to create and validate six distinct machine-learning algorithms to predict BRAF^V6OOE^ mutation in PTC patients prior to surgery.

## Materials and methods

### Patients

**Figure 1:**
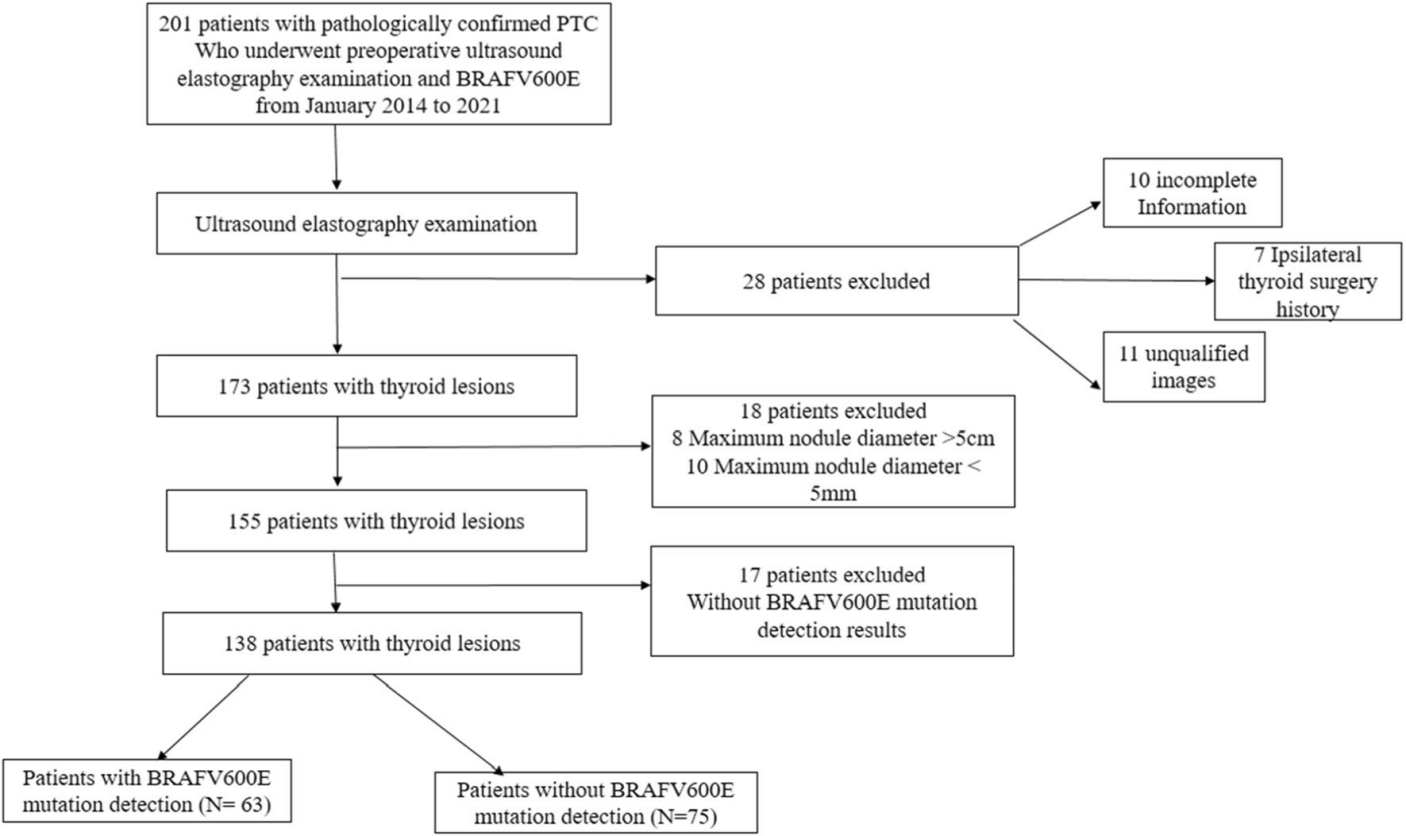
Schematic diagram of the patient selection. PTC, papillary thyroid carcinoma.

A retrospective analysis was performed on PTC patients who had undergone preoperative thyroid US elastography, BRAF^V600E^ mutation diagnosis, and surgery at Jiangsu University Affiliated People's Hospital and traditional Chinese medicine hospital of Nanjing Lishui District between January 2014 and 2021. The enrolling process is displayed in Fig. 1. 138 PTCs of 138 patients (mean age, 41.63 ± 11.36 [range, 25–65] years) were analyzed in this study. The patients were divided into BRAF^V600E^ mutation-free group (n = 75) and BRAF^V600E^ mutation group (n = 63). Using a stratified sample technique at a 7:3 ratio, all patients were randomly assigned to either the training group (n = 96) or the validation group (n = 42). The following criteria were required for inclusion: postoperative pathology indicated PTC; preoperative thyroid US elastography evaluation; related US images and diagnostic outcomes; maximum nodule diameter > 5 mm, and < 5 cm; and unilateral and single focal lesion. The exclusion criteria included a maximum nodule diameter of > 5 cm and indistinct US imaging of nodules caused by artifacts. The clinical details of the enrolled patients were documented, including age, sex, nodule diameter, nodule location, nodular echo, nodule boundary, nodule internal and peripheral blood flow, nodule elastic grading, calcification, CLNM, and BRAF^V600E^ mutation results. The Jiangsu University Affiliated People's Hospital and the traditional Chinese medicine hospital of Nanjing Lishui District Ethics Committee approved this study. Because it was retrospective in nature, it did not require written informed consent.

### Strain elastography ultrasound examination

There were two ultrasonic devices used: the Philips Q5 (both Healthcare, Eindhoven, Netherlands) and the GE LOGIC E20 (GE Medical Systems, American General) (L12-5 linear array probe, frequency: 10–14 MHz).

To acquire longitudinal and transverse images of the thyroid nodules, continuous longitudinal and transverse scanning was done while the patients were supine. Blood flow in and around the nodule, strain elastic grading of the nodule, calcification, and CLNM were all visible on the coexisting diagram, which also included the nodule diameter, location, echo, and boundary.

The cross-sectional image's position and size of the sampling frame were adjusted, and the strain elastic imaging mode was activated. With an ROI that was larger than the nodules (generally more than two times), the nodules were placed in the middle of the elastic imaging zone. Pressure was applied steadily (range 1–2 mm, 1–2 times/s) while the probe was perpendicular to the nodule. When the linear strain hint graph (green spring) suggested stability, the freeze key was pressed to get an elastic image; the ROI's color changed (green indicated soft; red indicated hard), and the nodule's hardness was determined based on elasticity. The elastic image was graded according to the following criteria: one point equals a nodular area that alternates between red, green, and blue; two points equal nodules that are partially red and partially green (mostly green, area > 90%); three points equal a nodule area that is primarily green, with surrounding tissues visible in red; four points equal a nodule area that is primarily red, with the red area > 90%; and five points equal a nodule area that is completely covered in red.

### Region of interests (ROIs) segmentation

**Figure 2:**
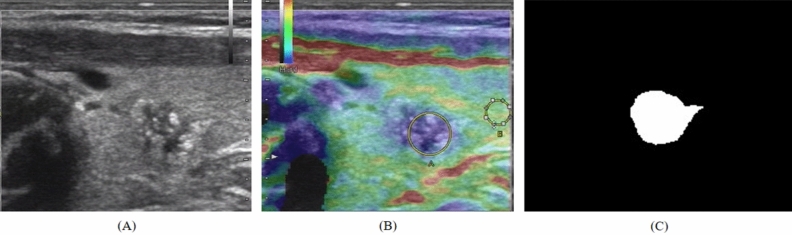
(**A**) Ultrasound conventional B-mode image of papillary thyroid carcinoma. (**B**) corresponding ultrasound elastography image, with the circle, labeled A indicating a lesion region and the circle labeled B indicating a reference area. (**C**) Corresponding image after region of interest (ROIs) segmentation step.

One week prior to surgery, thyroid US exams were conducted. US image segmentation was done manually. Using the ITK-SNAP program (http://www.itksnap.org), the ROIs were manually drawn on each image (Fig. 2). The grayscale images were used to create a sketch outline of the tumor regions in the elastography US images.

### Radiomic feature extraction

Radiomic features were extracted using PyRadiomics (https://github.com/Radiomics/pyradiomics). A total of 479 radiomic features were recovered from each ROI's elastography US images. Among those included were first-order Gray Level Co-occurrence Matrix (GLCM), Gray Level Run Length Matrix (GLRLM), Gray Level Size Zone Matrix (GLSZM), Gray Level Dependence Matrix (GLDM), and Neighbouring Gray Tone Difference Matrix (NGTDM) features, as well as features deduced from wavelet filter images containing first-order GLCM, GLRLM, GLSZM, GLDM, and NGTDM features.

### Radiomic feature selection

The retrieved features were normalized using a standard scalar to reduce bias and overfitting in the study. The dataset was divided into training and validation cohorts. To make each characteristic substantially independent, the row spatial dimension of the feature matrix was reduced using the Pearson correlation coefficient (PCC). Every pair of features with a PCC of more than 0.80 was deemed redundant.

After PCC, recursive feature elimination (RFE) for feature selection was applied to the whole dataset using the Scikit-learn python module^24^ to choose representative features for the training cohort. During the RFE procedure, the following parameters were taken into consideration (cross-validation was set to stratifiedkfold with the number of splits being 10, the random state was set to 101, minimum features to select was set to 3, and accuracy was employed for the scoring.

### Development of machine learning-based models

The Support Vector Machine with the linear kernel (SVM_L), Support Vector Machine with radial basis function kernel (SVM_RBF), LogisticRegression (LR), Naïve Bayes (NB), K-nearest Neighbors (KNN), and Linear Discriminant Analysis (LDA) classifiers were used to build the prediction models using the RFE’s key features. All six algorithms were implemented using the Scikit-learn machine learning library^24^

The same feature sets were chosen and fed into the model during the validation process. Standard clinical statistics like the area under the curve (AUC), sensitivity, specificity, negative predictive value (NPV), positive predictive value (PPV), and accuracy (ACC) were used to evaluate the model's performance on the training and validation datasets.

### Statistical analysis

Python (version 3.7, https://www.python.org/ Accessed 8 July 2021) and IBM SPSS Statistics (Monk Ar, New York, New York State, USA.) for Windows version 26.0 were used for statistical analyses. Pearson's chi-square and Fisher's exact tests were used to compare the differences in categorical characteristics. The independent sample t-test was used for continuous factors with normal distribution, whereas the Mann–Whitney U test was used for continuous factors without normal distribution.

A two‐sided P < 0.05 indicated statistically significant differences. PyRadiomics (version 2.2.0, https://github.com/Radiomics/pyradiomics Accessed 10 August 2021) and scikit‐learn version 1.2^24^ were used to extract radiomic features and build the prediction models. Each prediction model's AUC, sensitivity, specificity, ACC, NPV, and PPV were calculated.

Medcalc Statistical Software was used to calculate the six models’ AUCs and evaluate the predictions. The DeLong method was used to compare the AUCs of the six machine learning classifiers. To create calibration curves, the sci-kit-learn version 1.2^24^ was used. R software (version 3.6.1, https://www.r-project.org) was used to perform the decision curve analysis.

### Institutional review board statement

The study was conducted in accordance with the Declaration of Helsinki and approved by the Jiangsu University-Affiliated People’s Hospital and traditional Chinese medicine hospital of Nanjing Lishui District Ethics Committee.

### Informed consent

Patient consent was waived by the Jiangsu University-Affiliated People’s Hospital and traditional Chinese medicine hospital of Nanjing Lishui District ethics committee due to the retrospective nature of the study.

## Results

### Clinical characteristics

There were 138 PTC patients in all, 87 of whom were women (mean age, 41.81 ± 11.23 [range, 25–57] years), and 51 of them were men (mean age, 43.82 ± 12.18 [range, 28–65] years). In Table 1, 138 patients' clinical information and imaging comparisons between the training and validation groups are displayed.

**Table 1:** Comparison of clinical and ultrasonic characteristics of the PTC patients in the training and validation dataset.

Characteristic	Training cohort (n = 96)	Validation cohort (n = 42)	P-value
Age, mean ± SD (years)	41.78 ± 10.99	44.33 ± 12.81	0.152
Age (years)			
> 45	48.63 ± 5.23	49.90 ± 5.89	0.218
≤ 45	34.61 ± 5.17	34.76 ± 7.87	0.670
Sex			
Male	36	15	0.851
Female	60	27	
Tumor size(mm), mean ± SD	26.04 ± 8.51	26.63 ± 8.55	0.074
Primary site			
Right lobe	28	15	0.318
Left lobe	30	16	
Isthmus	38	11	
Tumor location			
Upper pole	31	18	0.325
Lower pole	27	13	
Middle	38	11	
Composition			
Solid	56	19	0.269
Predominantly solid	40	23	
Elastic classification			
1	13	5	0.375
2	22	15	
3	15	7	
4	26	7	
5	20	8	
Cystic change			
With cystic change	52	20	0.579
Without cystic change	44	22	
Calcification			
Microcalcification	37	11	0.143
Macrocalcification	41	17	
Rim calcification	18	14	
Tumor border			
Clear	37	13	0.510
Less clear	31	18	
Fuzzy	28	11	
Cervical lymph node metastasis	61	31	0.327

The relationship between the BRAF^V600E^ mutation and ultrasonic imaging characteristics and the predictive performance of the machine learning algorithms is shown in Tables 2, 3.

**Table 2:** Patient characteristics of the PTC with BRAF^V600E^ and PTC without BRAF^V600E^ groups.

	BRAFV600E(+) (n = 63)	BRAFV600E(−) (n = 75)	P-value
Age, mean ± SD, years	38.03 ± 10.41	36.68 ± 10.05	0.377
Sex			
Male	22	29	0.724
Female	41	46	
Tumor size, mean ± SD	24.12 ± 8.6	23.98 ± 11.01	0.928
Composition			
Solid	33	42	0.733
Predominantly solid	30	33	
Elastic classification			
1	2	1	0.015
2	2	0	
3	20	11	
4	14	13	
5	25	50	
Solid part echogenicity			
Markedly hypoechoic	41	20	0.000
Hypoechoic	10	21	
Isoechoic	9	14	
Hyperechoic	3	20	
Shape			
Irregular	32	43	0.443
Round to oval	31	32	
Vertical and horizontal diameter ratio			
≥ 1	41	27	0.001
< 1	22	48	
Margin			
Spiculated/microlobulated	30	25	0.183
Ill-defined	21	28	
Smooth	12	22	
Calcification			
Microcalcification	26	22	0.127
Macrocalcification	27	31	
Rim calcification	10	22	
FinalC-TIRADS category			
Low suspicion	16	31	0.058
Intermediate suspicion	17	22	
High suspicion	30	22	
Cervical lymph node metastasis	47	45	0.102

Clinical characteristics, such as age and gender, did not differ significantly between the two groups (P > 0.05). There were no significant differences in mean nodule size between the two groups (BRAF^V600E^ mutant group: 24.12 ± 8.6 mm; non-BRAF^V600E^ mutant group: 23.98 ± 11.01 mm, P = 0.928) or CLNM (P = 0.102). There was a significant difference in echogenicity (P = 0.000), vertical and horizontal diameter ratios (P = 0.001), and elasticity (P = 0.015) between PTC patients with BRAF^V600E^ and PTC patients without BRAF^V600E^.

**Table 3:** Predictive performance comparison of machine learning algorithms in the training and validation cohorts.

	ACC	AUC ± SE (95% CI)	SEN	SPEC	PPV	NPV
Training cohort						
KNN	0.89	0.96 ± 0.02 (0.89–0.99)	0.89	0.87	0.91	0.85
LDA	0.98	1.00 ± 0.00 (0.96–1.00)	0.98	0.97	0.98	0.97
LR	1.00	1.00 ± 0.00 (0.96–1.00)	1.00	1.00	1.00	1.00
NB	0.90	0.96 ± 0.02 (0.89–0.99)	0.90	0.90	0.93	0.85
SVM-L	1.00	1.00 ± 0.00 (0.96–1.00)	1.00	1.00	1.00	1.00
SVM-RBF	0.98	1.00 ± 0.00 (0.96–1.00)	0.96	1.00	1.00	0.95
Validation cohort						
KNN	0.81	0.87 ± 0.05 (0.73–0.95)	0.86	0.75	0.79	0.83
LDA	0.88	0.91 ± 0.05 (0.76–0.98)	0.91	0.85	0.87	0.89
LR	0.88	0.92 ± 0.05 (0.80–0.98)	0.91	0.85	0.87	0.89
NB	0.81	0.80 ± 0.08 (0.65–0.91)	0.82	0.82	0.80	0.80
SVM-L	0.88	0.93 ± 0.05 (0.80–0.98)	0.91	0.85	0.87	0.90
SVM-RBF	0.93	0.98 ± 0.02 (0.88–1.00)	0.95	0.90	0.91	0.95

AUC, area under the curve; ACC, accuracy; SEN, sensitivity; SPEC, specificity; NPV, negative predictive value; PPV, positive predictive value; USR, ultrasound radiomic; PTC, papillary thyroid carcinoma; SVM-L, support vector machine with linear kernel;SVM-RBF, support vector machine with radial basis function; KNN, K-nearest neighbour; NB, naïve bayes; LDA, linear discriminant analysis;LR, logistic regression.

### Diagnostic performance of the machine learning models in the training and validation cohorts

**Figure 3:**
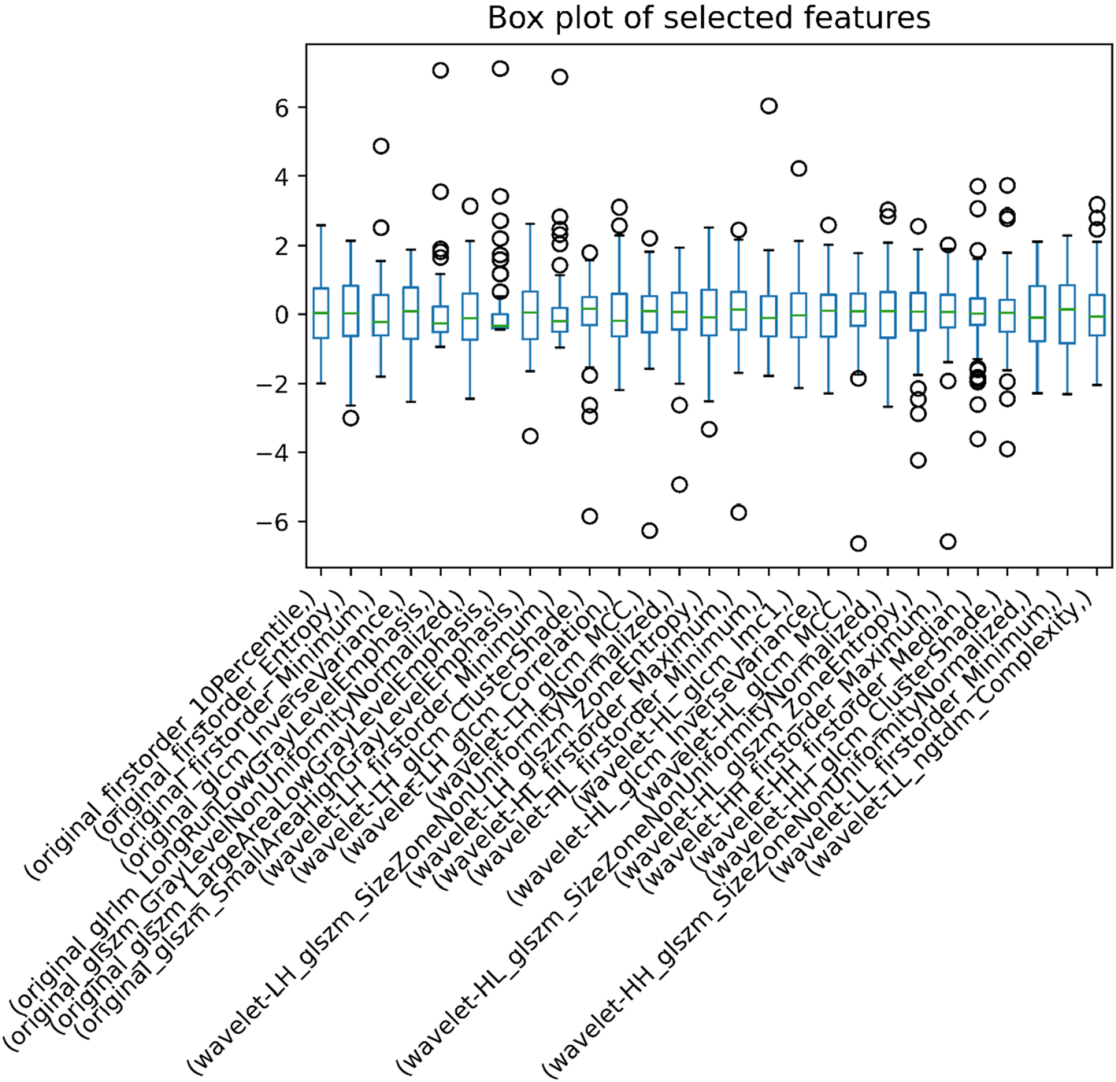
Boxplot of Selected features after RFE. Features were reduced to twenty-seven features during training of the machine learning models.

**Figure 4:**
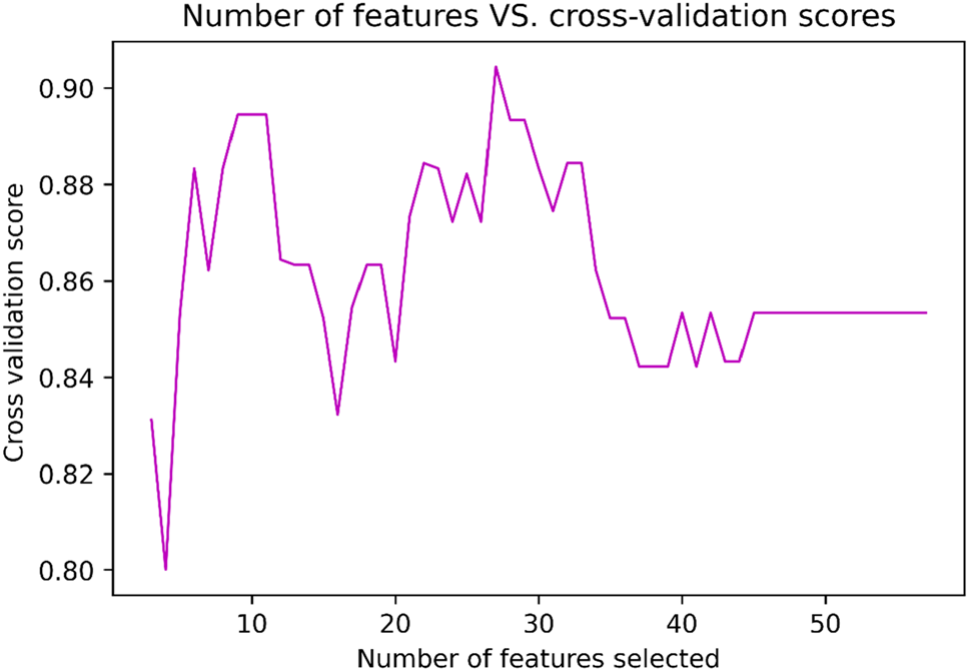
Recursive feature elimination (RFE) with tenfold cross‐validation; number of features selected vs. cross‐validation score.

After PCC and RFE with stratified tenfold cross-validation, 27 radiomic features were chosen in the training cohort (Figs. 3 and 4).

The following features were chosen to develop the predictive models for BRAF^V600E^ based on six machine learning algorithms (Table 4).

**Table 4:** Radiomics features selected after PCC and RFE analysis.

Radiomics features	Coefficient
original_firstorder_10Percentile	0.319091638765928
original_firstorder_Entropy	0.487563008130997
original_firstorder_Minimum	− 0.689397324848202
original_glcm_InverseVariance	0.320755497574734
original_glrlm_LongRunLowGrayLevelEmphasis	0.289249743498163
original_glszm_GrayLevelNonUniformityNormalized	− 0.462108224518231
original_glszm_LargeAreaLowGrayLevelEmphasis	1.53918767726622
original_glszm_SmallAreaHighGrayLevelEmphasis	− 0.4600168409863
wavelet-LH_firstorder_Minimum	0.203880750540529
wavelet-LH_glcm_ClusterShade	0.370856455050612
wavelet-LH_glcm_Correlation	− 0.527527316574072
wavelet-LH_glcm_MCC	− 0.628688962372851
wavelet-LH_glszm_SizeZoneNonUniformityNormalized	− 0.457585723931173
wavelet-LH_glszm_ZoneEntropy	− 0.699132116206135
wavelet-HL_firstorder_Maximum	0.749434461872676
wavelet-HL_firstorder_Minimum	− 0.790858716113758
wavelet-HL_glcm_Imc1	− 0.260391671132259
wavelet-HL_glcm_InverseVariance	0.58449015454453
wavelet-HL_glcm_MCC	0.672849806282339
wavelet-HL_glszm_SizeZoneNonUniformityNormalized	0.733532271458511
wavelet-HL_glszm_ZoneEntropy	0.686277806428273
wavelet-HH_firstorder_Maximum	− 0.532437149368159
wavelet-HH_firstorder_Median	0.334726274781584
wavelet-HH_glcm_ClusterShade	− 0.288808544114359
wavelet-HH_glszm_SizeZoneNonUniformityNormalized	0.359598216711341
wavelet-LL_firstorder_Minimum	0.268464336096376
wavelet-LL_ngtdm_Complexity	− 0.441332524522852

**Figure 5:**
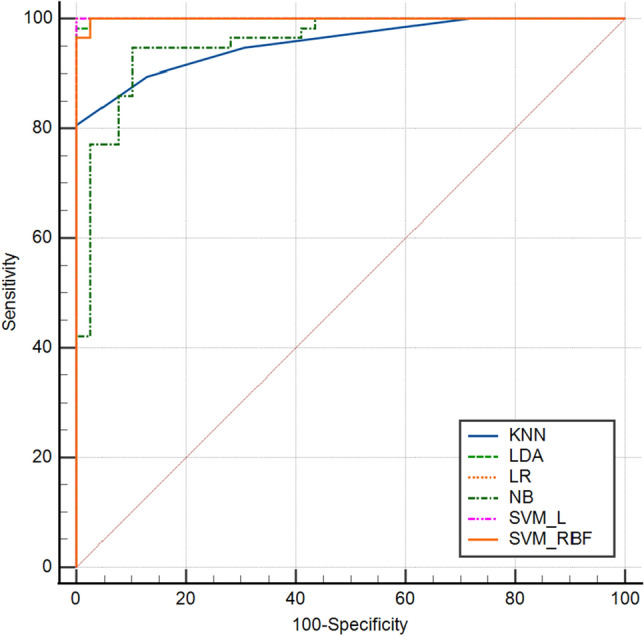
The mixed ROC curves of the six machine learning models in the training cohort. ROC: receiver operating characteristic; KNN: K-nearest neighbor; LDA: linear discriminant analysis; LR: logistic regression; NB: Naïve Bayes;SVM_L: support vector classifier with the linear kernel; SVM_RBF: support vector classifier with the radial basis function.

In the training cohort, AUCs for KNN, LDA, LR, NB, SVM_L, and SVM_ RBF were 0.96 (95% confidence interval [CI]: 0.89–0.99), 1.00 (95% CI 0.96–1.00), 1.00 (95% CI 0.96–1.00), 0.96 (95% CI 0.89–0.99), 1.00 (95% CI 0.96–0.1.00), and 1.00 (95% CI 0.96–1.00), respectively (Table 3 and Fig. 5). The SVM_RBF, SVM_L, LDA, and LR models performed best. KNN and NB followed. All machine learning models performed well.

**Figure 6:**
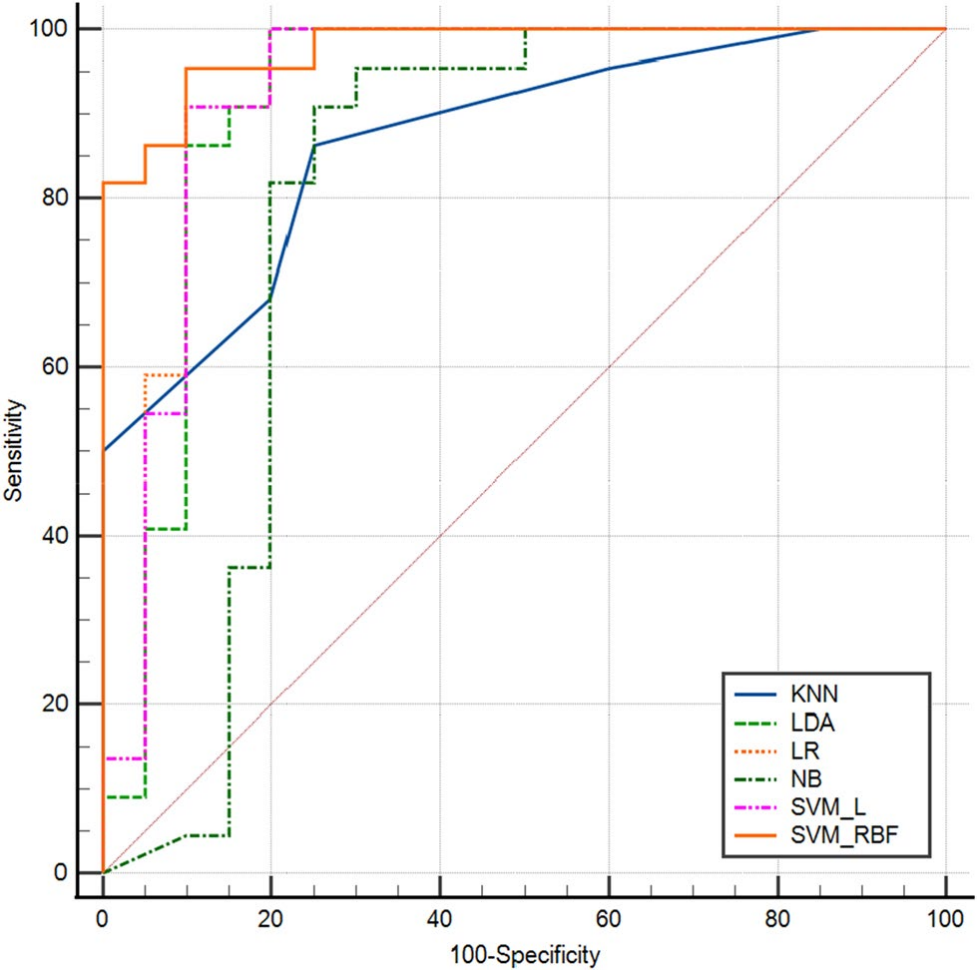
The mixed ROC curves of the six machine learning models in the validation cohort. ROC: receiver operating characteristic. KNN: K-nearest neighbor; LDA: linear discriminant analysis; LR: logistic regression; NB: Naïve Bayes; SVM_L: support vector classifier with the linear kernel; SVM_RBF: support vector classifier with the radial basis function.

In the validation cohort, AUCs for NB, KNN, LDA, LR, SVM_L, and SVM_RBF were 0.80 (95% confidence interval [CI]: 0.65–0.91), 0.87 (95% CI 0.73–0.95), 0.91(95% CI 0.79–0.98), 0.92 (95% CI 0.80–0.98), 0.93 (95% CI 0.80–0.98), and 0.98 (95% CI 0.88–1.00), respectively. The SVM_RBF model performed the best in the validation cohort, followed by the LR, SVM_L, LDA, KNN, and NB models, in that order (Fig. 6). All machine learning-based models performed well. The SVM_RBF model's sensitivity, specificity, PPV, and NPV were 0.95, 0.90, 0.91, and 0.95, respectively (Table 3).

**Figure 7:**
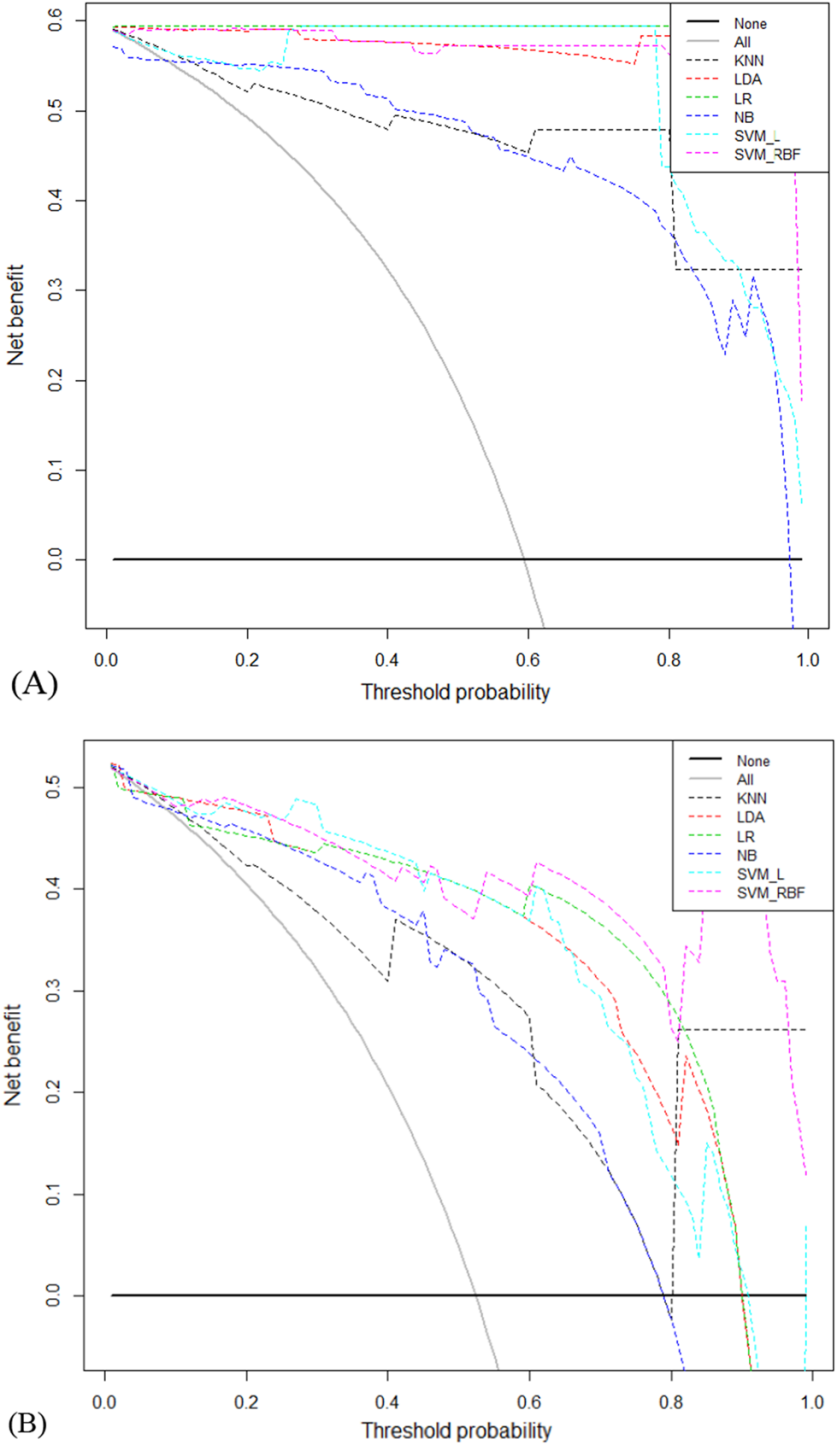
Decision curve for predictive models based on machine learning models in the training cohort (**A**) and the validation cohort (**B**). KNN K-nearest neighbor; LDA Linear discriminant analysis;LR Logistic regression; NB Naïve Bayes;SVM_L Support vector classifier with the linear kernel; SVM_RBF Support vector classifier with the radial basis function.

**Figure 8:**
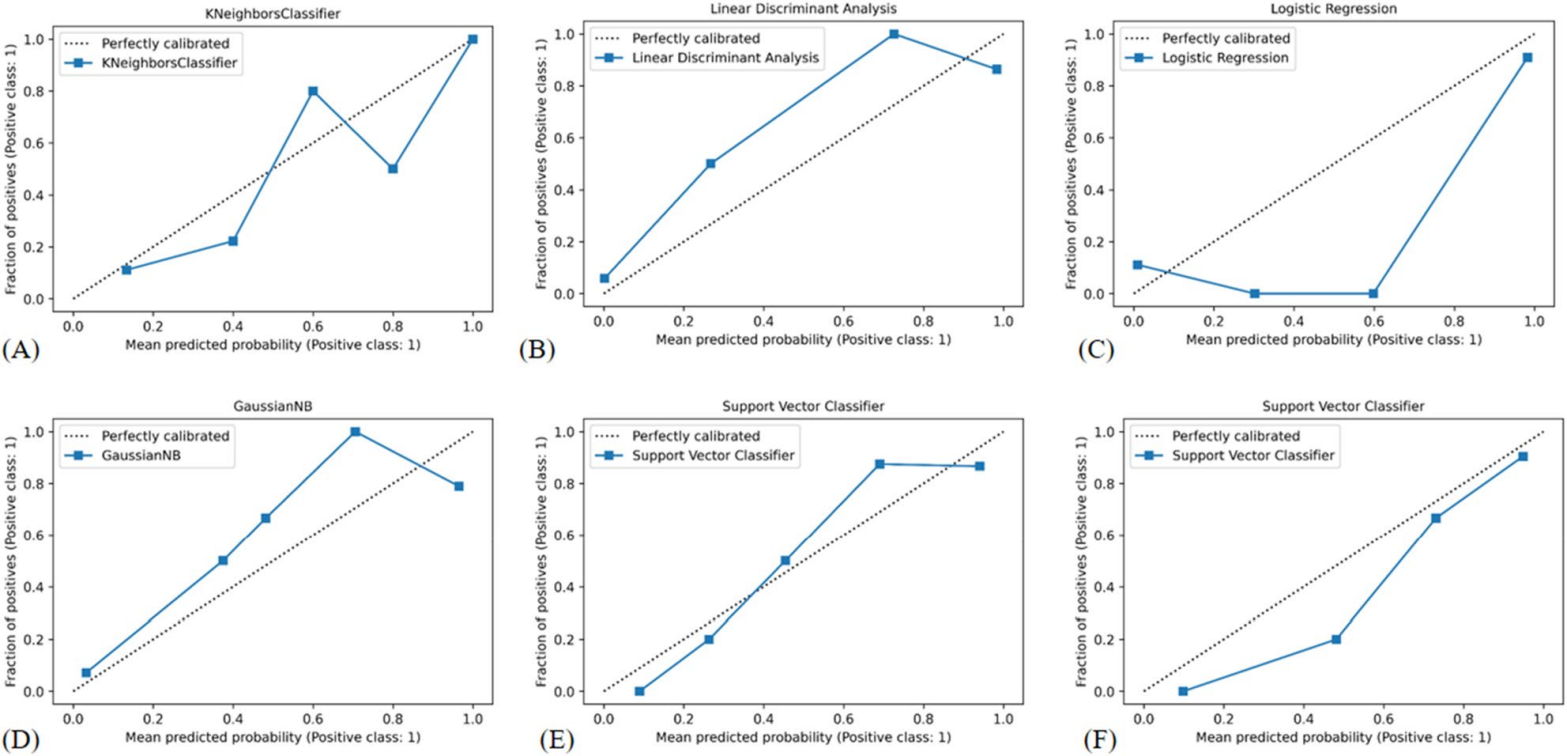
Calibration curves of the machine learning (**A**) K-nearest neighbor (**B**) Linear discriminant analysis (**C**) LR Logistic regression (**D**) Naïve Bayes (**E**) Support vector classifier with the linear kernel; (**F**) SVM_RBF Support vector classifier with the radial basis function.

Furthermore, the DCA was used to evaluate the clinical utility of these models (Fig. 7). The calibration curve was used for evaluating the probability accuracy of the machine learning models in predicting an individual outcome event in the future (Fig. 8).

## Discussion

The BRAF ^V600E^ mutation has become a distinctive and important molecular marker in the management of PTC due to its substantial relationship with aggressive clinical pathological outcome, serious molecular derangements, and selection of treatment methods in PTC.

Real-time tissue strain elastography helps assess the anatomical structure and biological traits of PTC because it represents the relative hardness of the lesion and its surrounding tissues, whereas the hardness of PTC tissues is closely related to its internal pathological structure. Bojunga et al.^25^ showed that elastography US could effectively distinguish between benign and malignant thyroid nodules. Recent research has demonstrated that varied cell arrangements and cell compositions result in varying tissue elastography indices^26,27^; the harder elastography US imaging is for PTC, the higher its malignancy. This gives the US diagnosis of BRAF^V600E^ mutation-positive and negative PTC a new viewpoint. Using US images, it was determined that patients with and without BRAF^V600E^ mutations had significantly different elastic moduli or levels of hardness. Elastography might more accurately describe the pathological features of tissues and directly and quantitatively indicate the absolute hardness of BRAF^V600E^ mutation-positive and -negative PTC. This study used machine learning methods to extract imaging omics features behind PTC strain elastic US images, and then used these features to build models to predict BRAF gene status, which is more objective than the semi-quantitative method of dividing PTC strain elastic US images into 1–5 points based on naked eye observation.

In a previous study^28^, we developed three radiomic models to predict BRAF^V600E^ mutation in PTC patients: a radiomic model based on grayscale US radiomic features, a radiomic model based on elastography radiomic features, and a third model based on combined gray US radiomic and elastography radiomic features using logistic regression classifier and the least absolute shrinkage selection operator (LASSO) feature selection algorithm. The gray scale US radiomic model did not perform well in the validation cohort (ACC: 0.67, AUC: 0.73), but the elastography model (ACC: 0.88, AUC: 0.93) and combined model (ACC: 0.91, AUC: 0.94), did perform well. Considering that elastic US images superimpose information on the hardness of the mass on the basis of grayscale US images, image feature extraction not only extracts the hardness characteristics of the mass, but also extracts rich features of the internal structure of the mass. So we decided to focus on elastography radiomics features in this current study to explore the performance of multiple machine learning classifiers or algorithms as well as other feature selection methods.

Machine learning algorithms benefit from learning from input data and automatically recognizing patterns and trends in that data automatically. There have been numerous studies^29–31^ on the use of machine learning to differentiate between benign and malignant thyroid nodules. However, there have been only a few studies using machine learning models to predict BRAF^V600E^ mutation in PTC patients. Additionally, there hasn't been a comparison of the effectiveness of various machine learning algorithms for the prediction of BRAF^V600E^ mutation in PTC patients based on US elastography radiomic features. In the current study, we constructed six machine-learning models to distinguish PTC patients with BRAF^V600E^ gene mutations from PTC patients without BRAF^V600E^ gene mutations using preoperative US elastography radiomic data. There were three noteworthy discoveries. First, based on preoperative US elastography radiomic features, the six machine learning models could differentiate PTC patients with BRAF^V600E^ from non-BRAF^V600E^ PTC patients. Second, when the six machine learning models were compared, SVM_RBF had the best prediction performance. Third the machine learning algorithms' diagnostic performance was based on 27 radiomic features.

In order to determine whether US-based radiomics could perhaps assess the occurrence of BRAF^V600E^ mutations among patients with PTC, Yoon et al.^10^ established a radiomic score using a dataset of 527 patients who had undergone surgical treatment for PTC and who had all undergone BRAF^V600E^ mutation analysis on surgical specimen.They reported that radiomics features extracted from US have limited value as a non-invasive biomarker for predicting the presence of BRAF^V600E^ mutation status of PTC, with a c-statistic value (equivalent to AUC) of 0.63 in the validation cohort and 0.72 in the training cohort. In comparison to this study, our study reported AUCs range of (0.96–1.00) and (0.87–0.98) in the training and validation cohorts, respectively, and ACCs range of (0.89–0.98) and (0.81–0.93) in the training and validation cohorts, respectively for the six machine learning algorithms employed in this study.

The higher AUCs and ACCs in the current study could be attributed to the US elastography radiomic features used, which provided more information than the grayscale US. Also in this study, machine learning algorithms were used in conjunction with radiomics, and several machine learning classifiers were used to build the models. To the best of our knowledge, this is the first study to develop models based on different machine-learning algorithms to predict BRAF^V600E^ mutations in PTC patients.

Comparing the current study results to our previous study^28^, the ACCs of SVM_L (0.88), LDA (0.88), and LR (0.88) were the same as the ACC of the logistic regression classifier (0.88) utilized in our previous study. However, the KNN (0.81) and NB (0.81) ACCs values were lower than the LR (0.88) method utilized in our previous study.

The AUCs of the KNN (0.87), LDA (0.91), LR (0.92), and NB (0.80) classifiers were all lower than the logistic regression classifier (0.93) utilized in our previous study. The SVM_L AUC (0.93) score, on the other hand, was the same as the logistic regression algorithm (0.93) utilized in our previous work. When compared to the logistic regression classifier (ACC: 0.88 and AUC: 0.93) employed in our previous investigation, the SVM_RBF had higher ACC (0.93) and AUC (0.98) values. The disparity in performance could be attributed to the various feature preprocessing techniques and feature selection strategies used in the current investigation.

On US examination, Kabaker et al.^32^ discovered that vertical and horizontal diameter ratios greater than one, as well as low echo, were all associated with the BRAF^V600E^ mutation. Similarly, Hahn et al.^33^ discovered that vertical and horizontal diameter ratios greater than one were linked to BRAF^V600E^ gene mutations. Consistent with these studies, we discovered in this current study that there was a significant difference in echogenicity, vertical and horizontal diameter ratios, and elasticity between PTC patients with BRAF^V600E^ and PTC patients without BRAF^V600E^.

The calibration curve of the prediction model is an essential metric for evaluating the probability accuracy of a disease risk model in predicting an individual outcome event in the future. A high degree of calibration shows that the prediction model is accurate, whereas a low degree of calibration indicates that the model may exaggerate or underestimate the risk of illness. The blue line reflects the performance of the machine learning algorithms, while the diagonal dotted line represents an ideal prediction (Fig. 8). A closer match to the diagonal dotted line suggests a better prediction. When the calibration curves were near the diagonal line, SVM_RBF, SVM_L, and NB algorithms demonstrated good agreement between the real status of BRAF^V600E^ gene mutation and the predicted probability.

Furthermore, the DCA was used to evaluate the clinical utility of these models (Fig. 7). Assuming that all patients do not have BRAF^V600E^ gene mutation, the solid black line (negative line) indicates that when no patient accepts intervention or treatment, the net benefit is zero. On the contrary, the solid grey line (positive line) indicates the net benefits when all patients have BRAF^V600E^ and receive treatments or interventions. According to the incidence of BRAF^V600E^ among patients with PTC, the reasonable range of thresholds was set from 0.3 to 0.99. In the entire range, all machine learning-based algorithms showed higher net benefits than the two extreme lines (negative line and positive line). In almost the entire threshold probability range, the SVM_RBF algorithm had the highest net benefit in both the training and validation cohorts (Fig. 7).

There are some limitations of the study, in the construction of the models to predict BRAF^V600E^ in PTC patients, the gene in healthy individuals was not analyzed, and the focus was on evaluating the BRAF^V600E^ mutation in PTC patients, which could have resulted in a selection bias. Also, this was a small sample retrospective study conducted at two institutions; thus, a selection bias may exist. In the future, we aim to conduct a multicenter study with a larger sample size.

## Conclusion

Finally, our study found that machine learning-based US elastography radiomic models performed well in predicting the potential of BRAF^V600E^ in PTC patients, which can assist physicians in identifying the risk of BRAF^V600E^ in PTC patients. SVM_RBF achieved the greatest prediction performance of the six machine learning models tested.

## Data Availability

The original contributions presented in the study are included in the article. Further inquiries can be directed to the corresponding authors.

## Abbreviations

ACC: Accuracy
AI: Artificial intelligence
AUC: Area under the curve
AUS/FLUS: Atypical lesions or follicular lesions of unknown significance
CLNM: Cervical lymph node metastasis
CSCO: Chinese Society of Clinical Oncology
DCA: Decision curve analysis
ETE: Extrathyroidal extension
FDA: Food and drugs authority
FNA: Fine needle aspiration
GLCM: Gray Level Co-occurrence Matrix
GLDM: Gray Level Dependence Matrix
GLRLM: Gray Level Run Length Matrix
GLSZM: Gray Level Size Zone Matrix
KNN: Knearest neigbor
LASSO: Least absolute shrinkage selection operator
LDA: Linear discriminant analysis
LR: Logistic regression
NCCN: National Comprehensive Cancer Network
NGTDM: Neighbouring Gray Tone Difference Matrix
NB: Naïve beyes
NPV: Negative predictive value
PCC: Pearson correlation coefficient
PPV: Positive predictive value
PTC: Papillary thyroid carcinoma
RFE: Recursive feature elimination
ROI: Receiver operating characteristics
SEN: Sensitivity
SPEC: Specificity
SUSP: Suspected malignant tumors
SVM_RBF: Support vector machine with radial basis function
SVM_L: Support vector machine with linear kernel
US: Ultrasound
